# Supplementary material for: Improving the Electrochemical Performance and Stability of Polypyrrole by Polymerizing Ionic Liquids
Source: Polymers (Basel). 2020 Jan 6;12(1):136. doi: 10.3390/polym12010136 (PMC7023371; doi:10.3390/polym12010136)
Supplement: Supplementary file 1 [file polymers-12-00136-s001.pdf]

Supplementary

Improving the Electrochemical Performance and Stability of Polypyrrole by Polymerizing Ionic Liquids

Arko Kesküla [1], Ivo Heinmaa [2], Tarmo Tamm [1], Nihan Aydemir [3], Jadranka Travas-Sejdic [3,4], Anna-Liisa Peikolainen [1] and Rudolf Kiefer [5,*]

[1] Intelligent Materials and Systems Lab, Institute of Technology, University of Tartu, Nooruse 1, 50411 Tartu, Estonia; arkokeskyla@gmail.com (A.K.); tarmo.tamm@ut.ee (T.T.); anna.liisa.peikolainen@ut.ee (A.-L.P.)

[2] National Institute of Chemical Physics and Biophysics, Akadeemia tee 23, 12,618 Tallinn, Estonia; ivo@kbfi.ee

[3] Polymer Electronics Research Center, School of Chemical Sciences, University of Auckland, Private Bag 92019, Auckland 1142, New Zealand; nayd972@aucklanduni.ac.nz (N.A.); j.travas-sejdic@auckland.ac.nz (J.T.-S.)

[4] MacDiarmid Institute for Advanced Materials and Nanotechnology, Wellington 6011, New Zealand

[5] Faculty of Applied Sciences, Ton Duc Thang University, Ho Chi Minh City 700000, Vietnam; rudolf.kiefer@tdtu.edu.vn (R.K.)

* Correspondence: rudolf.kiefer@tdtu.edu.vn; Tel.: +886-905605515

PIL Synthesis

All chemicals used for the preparation of the polymerizable ionic liquids were purchased from Sigma Aldrich and all solvents were HPLC grade. Figure S1 shows the synthesis of 1-vinyl-3-hexylimidazolium bromide (C6VImBr), prepared from the N-alkylation of 1-vinylimidazole (1 eq.) with 1-bromohexane (1.1 eq.).

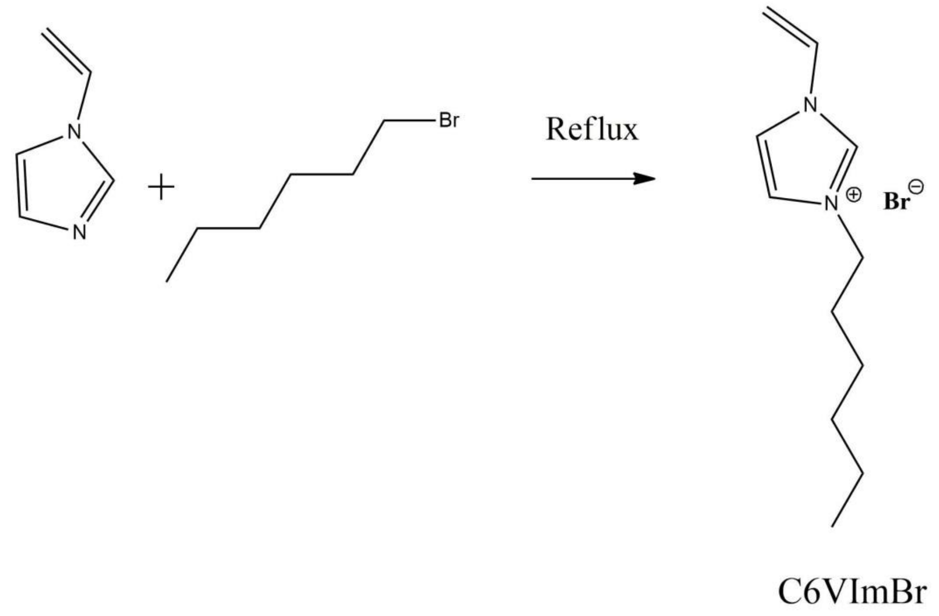

Figure S1. Synthesis of 1-vinyl-3 hexylimidazolium bromide (C6VImBr).

Under dry nitrogen atmosphere and vigorous stirring, 17.6 g of freshly distilled 1-bromohexane (106 mmol) was added drop-wise to 9.23 g of 1-vinylimidazole (98 mmol). The reaction mixture was left at 60 °C for 24 h. The mixture was cooled down to room temperature where two liquid phases separated. The upper phase was removed, and the remaining brownish ionic liquid 1-vinyl-3-hexylimidazolium bromide (C6VImBr) was washed three times with diethylether (3 × 30 ml) and then dried in a rotavapor. The product was further purified from an acetonitrile/diethylether mixture and finally dried under vacuum giving a 95.4% yield. The C6VImBr product was characterized by ^{1}H-NMR (400 MHz, DMSO, ppm) with signals appearing for ^{1}H-NMR at 8.95 (s, 1H), 7.65 (s, 1H), 7.45 (s, 1H), 7.13 (s, 1H), 7.1-7.07 (m, 1H), 5.79-5.75 (m, 1H), 4.21-4.17 (t, 2H), 1.89-1.81 (m, 2H), 1.28 (s, 6H) and 0.86-0.82 (m, 3H).

The final polymerizable ionic liquid 1-vinyl-3-hexylimidazolium TFSI (C6VImTFSI) was prepared by anion metathesis of (C6VImBr) using bis(trifluoromethylsulfonyl)imide lithium salt (LiTFSI) shown in Figure S2.

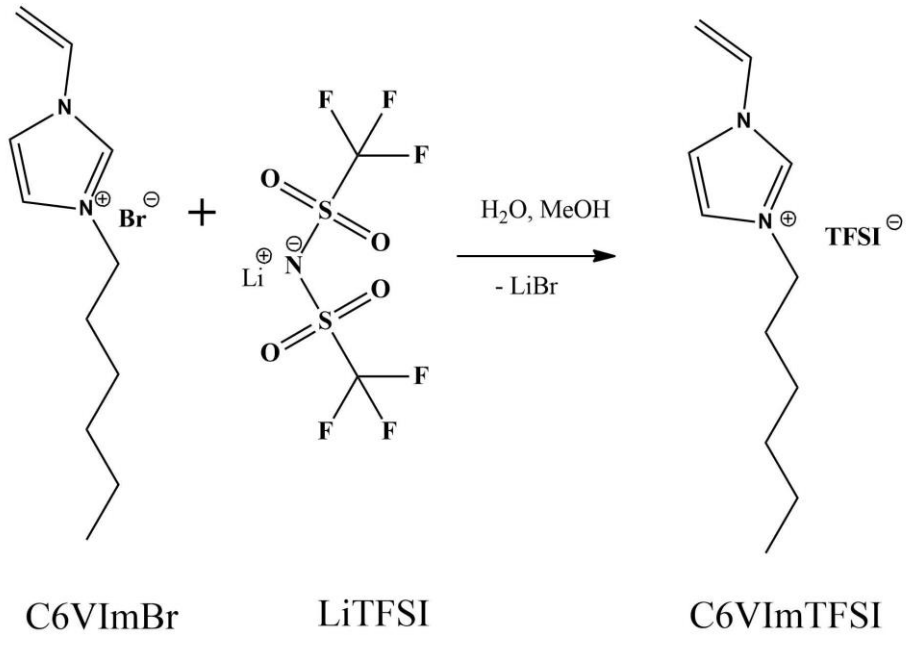

Figure S2. Anion exchange of PIL monomers C6VImBr to C6VImTFSI.

A solution of LiTFSI salt (3.58 g, 12 mmol) in 20 ml of deionized water was added dropwise to 3.16 g of 1-vinyl-3-hexylimidazolium bromide (12 mmol) dissolved in 60 ml of methanol. The reaction mixture was left at room temperature under stirring overnight and then concentrated under reduced pressure to remove methanol. After the removal of methanol, the separation of a brown viscous liquid occurred. The top layer was decanted and the remaining IL was washed three times with distilled water. The ionic liquid was then dried in a rotavapor at 50 °C.

Modified Scanning Ionic Conductance Microscopy (mSICM) Setup

Modified scanning ionic conductance microscopy [1] was developed in Auckland University to assist in conducting polymer micro fabrication [2], printing [3] and local characterizations [4].

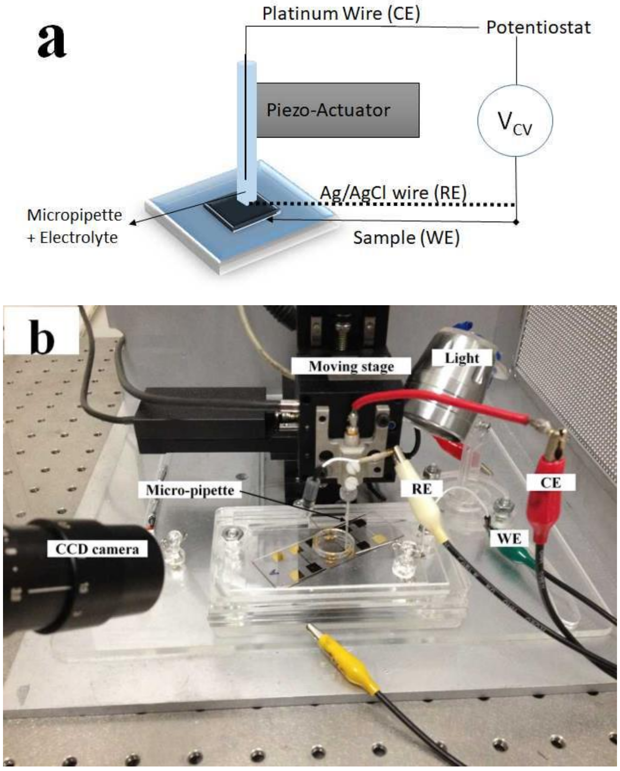

Figure S3. Setup of mSICM in **a**: Scheme of mSICM with samples (PPyPIL films in electrolyte) connected as working electrode (WE) with additional reference electrode (Ag/AgCl wire, RE). The micro-pipette can be positioned by a piezo actuator and contains the electrolyte, with the counter-electrode (CE, Pt wire) connected over the electrolyte droplet to the samples. **b**: Picture of the mSICM measurement set up with included CCD camera to fix the micropipette to the sample, and a light source.

Electrochemical Synthesis of PIL

Electrochemical synthesis of PIL was carried out galvanostatically (controlled with a PARSTAT 2273 potentiostat, Princeton Applied Research) in a two-electrode electrochemical cell at 0.1 mA/cm^2 for 40,000 s at −20 °C. The polymerization solution of PIL consisted of 0.1 M PIL monomer C6VImTFSI in propylene carbonate. Galvanostatic polymerization of PIL was carried out using a 15 mm x 40 mm stainless steel (AISI 316L) plate as the anode and stainless steel mesh (AISI 316L) as the cathode. The chronopotentiogram is shown in Figure S4.

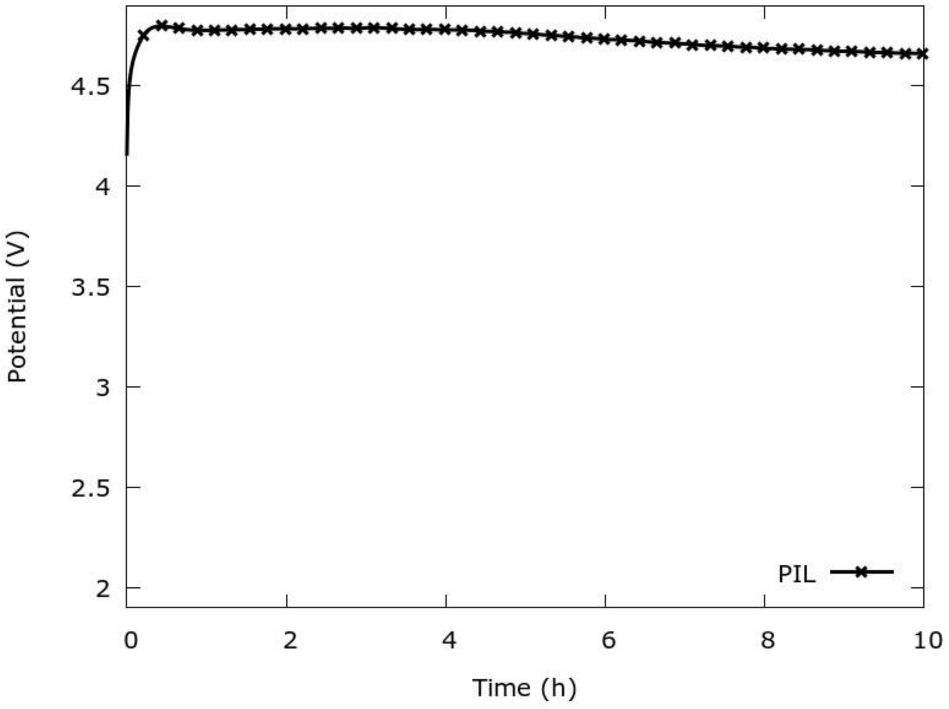

Figure S4. Chronopotentiogram of PIL synthesis.

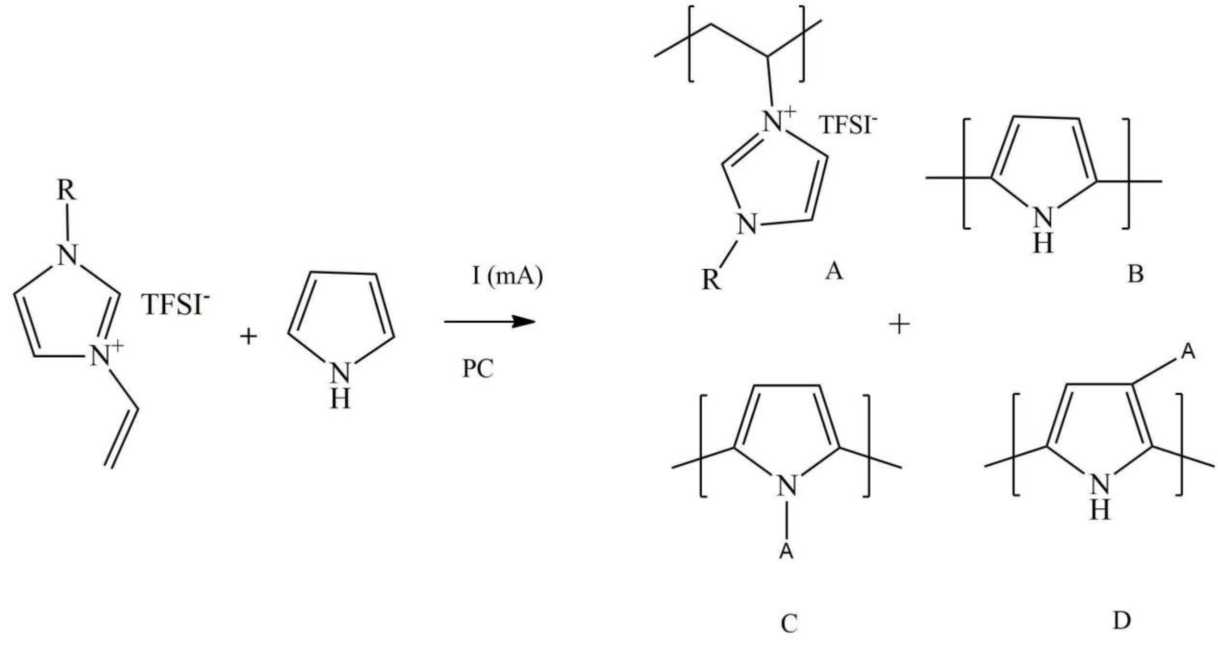

Figure S5. Structure of the proposed PPyPIL blends formed during electropolymerization of pyrrole and PIL monomer in PC with "R" representing the hexyl chain.

Electrochemical Measurements of Bulk PPyPIL Films

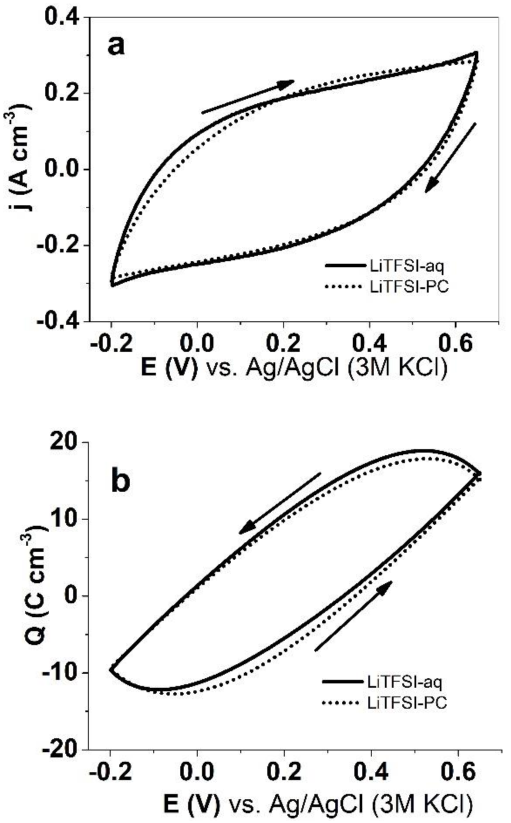

Figure S6. Cyclic voltammetry (5 mV s-1, 3rd cycle) measurements of PPyPIL films in LiTFSI-aq (solid line) and LiTFSI-PC (dotted) electrolytes (3 electrode cell: the PPyPIL sample as working electrode, a platinum sheet counter electrode and a Ag/AgCl (3M KCl) reference electrode) at potential range 0.65 to -0.2 V. The current density (j) curves vs. potential E are shown in (**a**) and the charge density Q vs. potential E are presented at (**b**). The arrows indicate the start and ending point of the cycles.

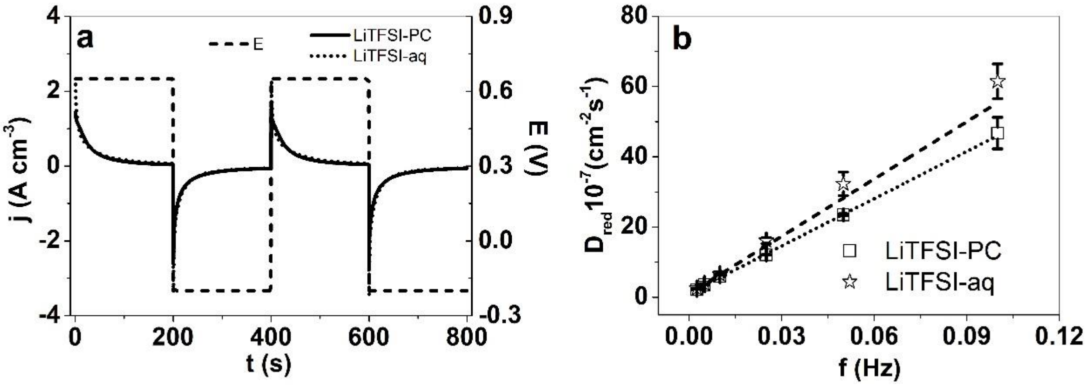

Figure S7. Square wave potential step measurements of PPyPIL films showing in (a): the current density time curves in LiTFSI-PC (line) and LiTFSI-aq (dotted) electrolytes in potential range 0.65 to -0.2 V (dashed line) at frequency of 0.0025 Hz (2nd and 3rd cycles). The diffusion coefficients D_{red} at reduction calculated from equation 1 and 2 are shown in (b) of PPyPIL films in LiTFSI-PC (□, dotted line as orientation) and LiTFSI-aq (☆, dashed line as orientation) at applied frequencies 0.0025 Hz to 0.1 Hz.

References

1. Laslau, C.; Williams, D.E.; Travas-Sejdic, J. Progress in Polymer Science The application of nanopipettes to conducting polymer fabrication , imaging and electrochemical characterization. *Prog. Polym. Sci.* **2012**, *37*, 1177–1191.
2. Laslau, C.; Williams, D.E.; Kannan, B.; Travas-Sejdic, J. Scanned Pipette Techniques for the Highly Localized Electrochemical Fabrication and Characterization of Conducting Polymer Thin Films , Microspots ,. *Adv. Funct. Mater.* **2011**, *21*, 4607–4616.
3. Aydemir, N.; Parcell, J.; Laslau, C.; Nieuwoudt, M.; Williams, D.E.; Travas-Sejdic, J. Direct Writing of Conducting Polymers. *Macromol. Rapid Commun.* **2013**, *34*, 1296–1300.
4. Laslau, C.; Williams, D.E.; Wright, B.E.; Travas-Sejdic, J. Measuring the Ionic Flux of an Electrochemically Actuated Conducting Polymer Using Modified Scanning Ion Conductance Microscopy. *J. Am. Chem. Soc.* **2011**, *133*, 5748–5751.
